# Urea Transporters in Cancer: Emerging Roles and Their Clinical Implications

**DOI:** 10.3390/biomedicines13112699

**Published:** 2025-11-03

**Authors:** Huimin Sun, Qiaoting Yang, Meng Ding, Shirui Li, Yi Xue

**Affiliations:** 1 Central Laboratory, Xiang’an Hospital of Xiamen University, School of Medicine, Xiamen University, Xiamen 361102, China; hmsun@xah.xmu.edu.cn; 2 School of Medicine, Xiamen University, Xiamen 361004, China; 3 Department of Dermatology, Children’s Hospital, Zhejiang University School of Medicine, National Clinical Research Center for Child Health, Hangzhou 310052, China

**Keywords:** *SLC14A* family, urea transporters, cancer

## Abstract

**Background/Objective:** The *SLC14A* gene family in mammals encodes the urea transporters UT-A and UT-B (UTs), whose primary function is urea transport. In recent years, increasing research has shown that UTs are involved in tumor formation and progression. The purpose of this article is to review the current knowledge in the potential of urea transporters as tumor biomarkers and therapeutic targets. **Methods:** An extensive review of the literature was performed utilizing both PubMed and Web of Science databases focusing on articles published within the last ten years. **Results:** UTs are significantly downregulated in various tumor tissues. They are associated with the staging and prognosis of cancers such as bladder cancer (BC). They participate in the metabolic reprogramming of tumor cells via glycosylation, enhancing the energy supply and material synthesis of tumor cells. They participate in remodeling the tumor microenvironment, influencing the interactions between fibroblasts and tumor cells. Additionally, they interact with other signaling pathways to participate in tumorigenesis and development. **Conclusions:** The mechanisms by which UTs function in tumorigenesis and progression remain to be fully elucidated, but their potential as tumor biomarkers and therapeutic targets has gradually attracted attention.

## 1. Introduction

Cellular metabolism and bioenergetic homeostasis are fundamental to oncogenesis and malignant transformation. The accelerated growth and sustained viability of neoplastic cells necessitate substantial nutrient acquisition. Aberrant metabolic reprogramming frequently leads to pathological alterations in ATP generation and macromolecular synthesis, representing key oncogenic characteristics [1]. Elucidating these mechanisms provides critical insights for developing innovative anticancer strategies.

Urea metabolism and its associated transport systems play critical physiological roles. As the primary nitrogenous waste product, urea contributes significantly to nitrogen homeostasis. Urea transporters mediate transmembrane urea movement, a process essential for renal function and fluid regulation. In oncological contexts, these transporters potentially modulate the tumor microenvironment and cellular activities, while malignant cells may exhibit dysregulated urea cycle activity to support specific biosynthetic pathways [2]. Research has revealed that neoplastic cells can reprogram urea metabolic pathways for energy production, with urea transport mechanisms serving pivotal functions in this process [3].

Urea serves as the principal nitrogenous waste product in mammalian metabolism, with its cellular transport predominantly facilitated by urea transporters (UTs) belonging to the SLC14A gene family [4]. These specialized membrane proteins play a crucial role in urea translocation, thereby facilitating water conservation and urinary concentration through the establishment of an osmotic gradient in the renal medulla. Beyond renal function, UT isoforms expressed in extrarenal tissues including cerebral tissue, testicular tissue, and vascular endothelium contribute significantly to regional osmoregulation and nitrogen homeostasis maintenance.

UTs are differentially expressed in tumor and normal tissues. By regulating urea metabolism and osmotic balance, urea transporters participate in the metabolic reprogramming of tumor cells, affecting the osmotic pressure, pH, and metabolite distribution in the microenvironment, thereby altering tumor cell proliferation, migration, and invasion [5]. They also interact with other signaling pathways in tumor energy metabolism and stress responses, driving tumor progression, and their potential as therapeutic targets has garnered attention [6].

However, there is a knowledge gap regarding the role of urea transporters in cancer. Despite the increasing understanding of cancer metabolism, the specific functions and regulatory mechanisms of urea transporters in tumor cells remain largely unknown. Further research is required to explore their potential as diagnostic markers or therapeutic targets. This review summarizes the reported research on the association between urea transporters and tumorigenesis, categorizing their roles in tumor development, aiming to inspire cancer researchers and highlight the significance of this family in tumor studies.

## 2. Materials and Methods

We performed an extensive literature review utilizing PubMed (https://pubmed.ncbi.nlm.nih.gov/) accessed on 15 July 2025, and Web of Science (https://www.webofscience.com/) databases, accessed on 16 July 2025, respectively. Our search strategy incorporated key terms such as “*SLC14A* family”, “urea transporters”, “tumorigenesis”, “clinical relevance”, and “single nucleotide polymorphisms”. The search was restricted to English-language publications from the previous decade (2015–2025) involving human subjects. The initial database query yielded 151 publications. Following title and abstract evaluation, 133 full-text articles underwent detailed eligibility assessment. Final inclusion criteria encompassed studies examining SLC14A gene single nucleotide polymorphisms in relation to tumors, epigenetic alterations of urea transporter proteins, alternative splicing variants and tissue distribution of UT proteins, clinical significance of UT proteins in oncology, and molecular mechanisms of UT proteins in cancer progression. We excluded non-English publications and studies published more than ten years prior to our search. Through thematic analysis of the selected 102 studies, we synthesized current evidence and emerging patterns concerning the clinical implications of urea transporters in malignant diseases and their prospective roles in cancer management and prevention strategies.

## 3. Results

### 3.1. Molecular Structure, Tissue Distribution, and Physiological Functions of UTs

**Figure 1:**
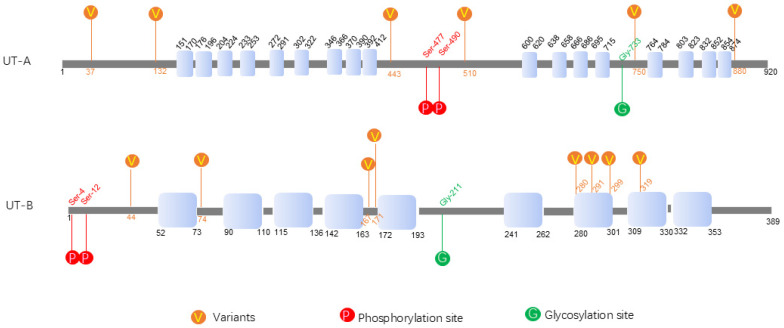
The structures of UT-A and UT-B proteins, their domains, and the sites of glycosylation, phosphorylation, and variation are, respectively, displayed. V represents variable sites. P represents phosphorylation sites. G represents glycosylation sites. (The data are from UNIPROT, https://www.uniprot.org, accessed on 18 July 2025).

The SLC14A1 gene encodes UT-B, which has a relatively simple gene structure with 2 splice variants (encoding UT-B1 and UT-B2). The SLC14A2 gene encodes UT-A, which has a complex gene structure with multiple exons and 6 identified splice variants [7] (Figure 1). Both UT-A and UT-B exhibit typical urea transporter characteristics on structure [8]; however, they differ in functional domains [7,9]. UT-A contains multiple transmembrane domains, with its N- and C-termini located intracellularly and the middle portion forming the urea channel. UT-A1 and UT-A2 are the most extensively studied isoforms. They are generated through alternative splicing, sharing partial coding sequences, but displaying distinct tissue distributions and functional properties. The structure of the UT-B protein is simpler than that of UT-A, typically composed of 10 transmembrane helices, with its urea channel located between the 3rd and 4th transmembrane helices [10]. Information of all known urea transporters was listed in Table 1.

**Figure 2:**
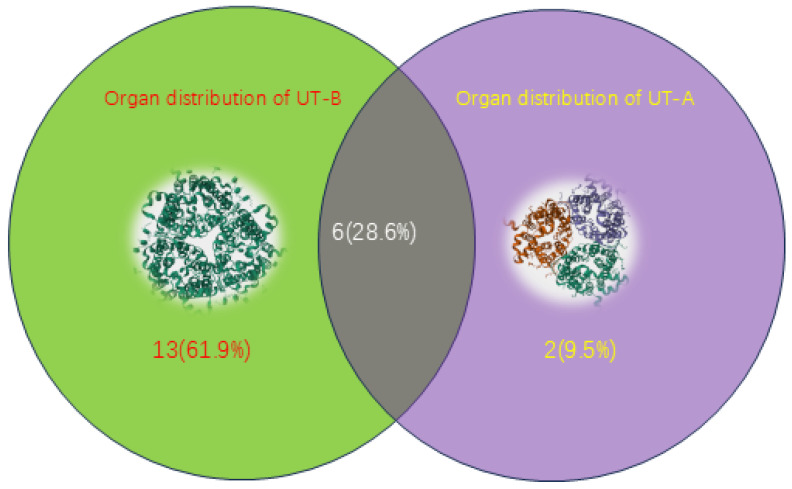
The three-dimensional structure of UT-A (**right**) and UT-B (**left**), Venn diagrams of the organs where UT-A and UT-B are mainly distributed. UT-A is mainly distributed in kidneys, hearts, brain, liver, large intestine, testes, ears and eyes; UT-B is mainly distributed in erythrocyte, endothelium, kidneys, colon, small intestine, cecum, heart, brain, spleen, testicles, bone marrow, prostate, bladder, thymus, skeletal muscle, lungs, pancreas, gastric glands andeyes. (The structure of proteins UT-A and UT-B was obtained from UNIPROT, https://www.uniprot.org, accessed on 13 July 2025). Venn diagram was performed by venny2.1 (https://bioinfogp.cnb.csic.es/tools/venny/, accessed on 26 October 2025).

Urea transporters are specifically distributed in certain tissues and organs, enabling them to become contributing factors for tumorigenesis in these organs (Figure 2). UT-A exhibits a highly region-specific distribution in the kidney [11], where UT-A1 and UT-A2 jointly participate in the urine concentration mechanism, maintaining the medullary hypertonic environment through the countercurrent multiplication system [12]. Besides the kidney tissue, UT-A is also expressed in the brain, testes, and vascular endothelium. In brain tissue, UT-A influences neuronal and glial cell function by regulating urea concentration. UT-A2 in rat is localized only in the kidney. UT-B is highly expressed in tissues such as erythrocyte membranes, brain, and testes. UT-B1 in rat is found in the brain, spleen, kidney, and testes [4,13]. Multiple studies indicate that UT-B is expressed in the bladder urothelium [13], where it is strongly expressed in all urothelial layers except the apical membrane of the outermost umbrella cells [13]. At the RNA level, UT-B1 is strongly expressed, while UT-B2 is only weakly present. Research by Stella Koutros et al. shows that SLC14A2 is primarily expressed in mammary and kidney tissues but is undetectable in the bladder. SLC14A1 is expressed in various tissues, including the bladder, prostate basal-type cells, and brain, with weaker expression detected in the kidney [14]. The organ-specific expression pattern of the urea transporters enables them to serve as biomarkers for abnormal situations such as tumorigenesis. These transporters can reflect the physiological and pathological states of specific organs. Therefore, their expression changes under abnormal conditions like tumor formation, and these changes can be detected and used as an indicator.

**Table 1:** The Informations of all known urea transporters.

Gene	Isoforms	Ecoded by Exons	Number of AA	Specific Motif or Mutation	Transmembrane Helices	Protein Expression in Normal Tissue	Function	References
*SLC14A1*	UT-B1	4–11	389	ALE motif	10	Human/Rat/Mouse liver, colon, testis, brain, heart, lung, aorta, cochlea, spinotrape zius muscle, urothelial, and mesenteric artery	Facilitate the permeation of urea	[4,8,11,13]
	UT-B2		389		10	Sheep and cow rumens		
*SLC14A2*	UT-A1	1–12, 14–23	929		20	Apical plasma membrane and in the cytoplasm of the IMCD in rat, mouse, and human medulla.	Urinary concentrating mechanism	
	UT-A2	13–23	397	Val/Ile 227, Ala/Thr 357	19	Thin descending limb of the loop of Henle	Minor role under physiologic conditions	
	UT-A3	1–12	460	glycosylation site at N279		Basolateral membrane of principle cells in inner medullary collecting ducts (IMCDs)	Facilitates transepithelial urea transport across the IMCD	
	UT-A4	1–7, 18–23	466	glycosylation site N 223		Rat kidney outer or inner medulla (tubular location is uncertain)	Could activated by cyclic AMP	
	UT-A5		323			Outermost layer of the seminiferous tubules inside the testes (mouse)	corresponds with the stage of testicular development	
	UT-A6	1–11	235			Human gastrointestinal system, especially in the colon	Expression is controlled by the contents of the intestinal mucosa.	

#### Genetic Variations Associated with Tumors

SNPs in genes often lead to functional changes and are major risk factors for carcinogenesis [15]. 16 SNPs in the SLC14A1 gene have been reported to be associated with tumor susceptibility, especially bladder cancer susceptibility (Table 2). rs1058396 is a high-risk variant in SLC14A1 [AG, GG], and it increases bladder cancer risk, particularly in never-smokers [16,17,18]. This locus harbors two variants, D280Y and D280N, both of which were predicted to significantly affect UT-B1 protein stability, reduce its binding affinity with ligands, and impair its function [19]. The rs17674580 [C/T] variant has been demonstrated to be associated with bladder cancer susceptibility in cohorts from European Caucasian populations, Japanese, Chinese, and northern Indian populations [18,20,21,22]. Analysis of urine-specific gravity (USG) revealed that rs10775480 is also correlated with increased bladder cancer susceptibility [14,23].

Except for SNPs, other types of variants in the SLC14A1 gene and UT-B protein have also been found to be associated with the increase in bladder cancer. Guo et al. detected SLC14A1 gene rearrangements and reported aberrant splicing of SLC14A1 in patients with bladder urothelial carcinoma [24]. UT-B protein owns two isoforms. The long isoform UT-B2 is expressed in normal bladder tissue, but lost in 33% of bladder cancers and significantly downregulated in 67% of patients. The short isoform UT-B1 (lacking exon 3) is present in 55% of bladder tumors, while another 55% of bladder tumors exhibit an in—frame deletion of 24 nucleotides in exon 4 of UT-B1 (UT-B1Δ24) [25].

SLC14A2 has been reported to have 9 tumor-associated SNPs, which are associated with susceptibility to various tumors (Table 2). rs9952980 is located in an intron of SLC14A2, and it was predicted to be able to regulate the expression of UT-A [26]. Recently, rs9952980 was discovered to affect the fifth nucleotide of ERE and was verified to significantly reduce ESR1 affinity. Compared to the wild type, SNP rs9952980 is sufficient to abolish the enhancer activity at this genomic locus. Additionally, according to TCGA data, rs9952980 was found to be significantly associated with reduced SLC14A2 expression in breast cancer patients, likely mediated by its direct impact on ESR1-DNA binding [27]. rs2243803 is another SNP that was found to be associated with a higher risk of breast cancer [28]. Jenni Hällfors et al. identified several SNPs in SLC14A2 associated with nicotine addiction [29]. Nicotine addiction has a strong correlation with cancers, such as lung cancer and head and neck cancer [30]. Several other SNPs in SLC14A2 gene were reported to be associated with esophageal squamous cell carcinoma [31], neurofibromatosis [32], follicular lymphoma [33], breast cancer [34] and bladder cancer [35], etc. This variant may influence kidney function and urine concentration, which subsequently become a risk factor of bladder cancer.

**Table 2:** Cancer-related SNPs on the SLC14A family genes.

Genes	SNP	Mutation	Cancers	References
*SLC14A1*	rs10853535	C>A,G,T	urinary bladder cancer	[16,18,25]
	rs1058396	G>A,C,T		
	rs11877062	C>T		
	rs17674580 rs11082469	C>A,T		
	rs11082469 rs11877720	A>G		
	rs11877720	A>A,G		
	rs10432193	T>C	bladder cancer	[16,19,23]
	rs9304322	G>C,T		
	rs2170974	A>G,T		
	rs9967412	C>A,G		
	rs10460035	G>A		
	rs8099449	T>C,G		
	rs12454680	T>A,C		
	rs568418	A>G,T		
	rs10775480	T>A,C		
	rs7238033	T>C	urothelial cancer	[36]
*SLC14A2*	rs9952980	T>A,C	breast cancer	[27,28]
	rs12455117	A>C,T		
	rs2243803	T>A		
	rs145751449	G>T	neurofibroma	[32]
	rs41301139	G>A,T		
	rs11082438	G>T	follicular lymphoma	[33]
	rs117354958	A>G	bladder cancer	[35]
	rs78012883	A>G		
	rs112833408	C>T		

### 3.2. Tumor-Associated Post-Translational Modification (PTM) Regulatory Mechanisms of UTs

Post-translational modifications (PTMs) significantly affect protein functionality and are essential in a wide range of cellular biological processes [36]. The intricate array of PTMs and their interactions are linked to numerous pivotal signaling events that contribute to tumor transformation, oncogenesis, and metastasis [37]. The pathological implications of different PTMs encompass various characteristics of cancer, including hallmark functions, metabolic alterations, and the regulation of the tumor microenvironment [38,39].

#### 3.2.1. Phosphorylation and Ubiquitination Modifications of UTs

Phosphorylation modification, the most common alteration in tumor cells, often leads to protein dysfunction and promotes tumor formation and progression [40,41]. PKA phosphorylates UT-A1 at S486 and S499 sites [42]. Studies have shown that elevated cAMP enhances PKA-mediated phosphorylation at these two sites, and this is a process that does not involve the vasopressin-sensitive exchange protein (Epac) activated by the cAMP pathway [43].

Phosphorylation and ubiquitination modifications are often interrelated, exhibiting both synergistic and antagonistic effects, which together precisely regulate protein function, localization, and degradation, thereby maintaining cellular homeostasis and responding to external stimuli. The PKA-cAMP pathway is widely involved in tumor progression, invasion, neuroendocrine transformation, distant metastasis, and metabolism, ultimately promoting tumor drug resistance [44]. 14-3-3γ interacts with the E3 ubiquitin ligase MDM2, increasing UT-A1 ubiquitination and degradation, thereby reducing urea transport [45]. Activation of cAMP/PKA enhances the interaction between 14-3-3γ and UT-A1 and stimulates MDM2-mediated ubiquitination and degradation of UT-A1, forming a novel regulatory mechanism for urea transport activity. Forskolin (FSK) activates the cAMP/PKA pathway, which leads to UT-A1 monoubiquitination on the cytoplasmic membrane, promoting UT-A1 endocytosis and accumulation in early endosomes. The phosphorylation of Ser486 and Ser499 sites of UT-A1 by PKA is essential for FSK-induced UT-A1 monoubiquitination. FSK activation of UT-A1 triggers UT-A1 monoubiquitination and subsequent lysosomal protein degradation [46]. UT-A1 could be phosphorylated by PKCα at S494, enhancing vasopressin-stimulated urea transport [47]. In rat inner medullary collecting ducts (IMCD), UT-A1 can be directly phosphorylated by AMPK. Metformin, an AMPK activator, can also phosphorylate UT-A1 [48].

**Figure 3:**
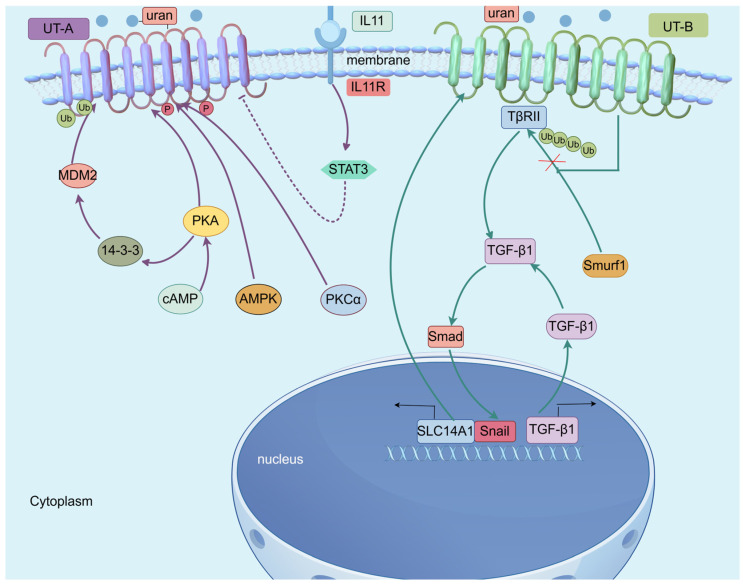
Crosstalk of UTs with other signaling pathways. UT-A can be directly phosphorylated by PKA, AMPK, and PKC and can be ubiquitinated by MDM2. IL-11R inhibits UT-A via regulating STAT3. UT-B interacts with TβRII and stabilizes the TβRII protein, hindering Smurf1 mediated K48 linked ubiquitination degradation and enhancing TGF-β/Smad signaling. Snail directly upregulates SLC14A1 via transcriptional regulation. TGF-β pathway and SLC14A1 establish a positive feedback loop. The figure was created by Figdraw (www.figdraw.com, accessed on 3 August 2025).

Only a few studies about the phosphorylation and ubiquitination of UT-B have been reported. A recent study on CRC has shown that UT-B interacts with TβRII and stabilizes the TβRII protein, hindering Smurf1-mediated K48-linked ubiquitination degradation and enhancing TGF-β/Smad signaling. Additionally, Snail directly upregulates SLC14A1 via transcriptional regulation. and thus establishes a positive feedback loop between TGF-β pathway and SLC14A1 [49] (Figure 3 and Table 3).

#### 3.2.2. Glycosylation Modification

Glycosylation is a key mode of protein modification in organisms, playing a crucial role in regulating various biological functions by influencing protein folding, transport, and localization [50]. Glycosylated proteins such as HER2, AFP, CEA, and PSA are approved tumor biomarkers clinically [51,52]. Alterations in glycosylation patterns are a significant hallmark of cancer [53,54,55]. Both UT-A and UT-B possess glycosylation sites and can undergo glycosylation modifications.

UT-A1 has two glycoforms, 117 kD and 97 kD, respectively [56,57]. The increased abundance of the highly glycosylated 117-kD UT-A1 corresponds to enhanced tubular urea permeability. Activation of PKC via Src kinase specifically promotes UT-A1 glycan sialylation in UT-A1-MDCK cells and rat renal inner medullary collecting duct suspensions, further elevating urea transport activity. UT-A3 comprises only the NH (2)-terminal half of UT-A1 and exists in two glycosylated forms of 45 kDa and 65 kDa, with its known glycosylation site at Asn279. The highly glycosylated UT-A3 is stably and highly expressed in lipid raft domains on the cell membranes of renal inner medullary cells, playing a crucial role in urea reabsorption [58]. Loss of glycosylation reduces UT-A3 membrane expression and urea transport activity, while mature UT-A3 glycans contain abundant sialic acid. The sialyltransferase ST6GalI catabolizes UT-A3 α2,6-sialylation. The activation of Protein Kinase C (PKC) promotes the sialylation of UT-A3 glycans and enhances its expression on the membrane surface. The enzyme ST6GalI facilitates this sialylation process, which in turn contributes to the stabilization of the UT-A3 protein and urea transport activity [59].

The UT-B protein also contains glycosylation sites. Walpole et al. detected a distinct signal corresponding to 40 to 45 kDa UT-B in human bladder proteins through the application of a novel UT-B COOH-terminal antibody. The use of peptide-N-glycosidase F enzyme effectively deglycosylated this UT-B signal, resulting in a core protein of 30 kDa, which is notably smaller than the anticipated size of UT-B1 [60]. A 35 kDa glycosylated UT-B protein was identified in human colonic mucosa [58]. The bladder cancer-associated UT-B1Δ24 exhibited minimal sialylation, suggesting impaired glycosylation of UT-B1 in bladder tumors. Altered expression of UT-B could potentially facilitate the advancement of bladder cancer or amplify the carcinogenic effects triggered by additional carcinogens. [25].

Glycosylated urea transporters interact with many protein kinases to regulate physiological functions. In various tumors, protein kinases such as SRC and PKC are often in a dysregulated state due to bypass activation, and thus glycosylated urea transporters will also change accordingly, showing dysfunction.

#### 3.2.3. Methylation Modification

Methylation is an important type of epigenetic regulation that depends on the catalysis of methyltransferase. In tumors, methylation dysregulation plays a role in promoting tumor progression in multiple aspects [61,62,63,64].

Ma et al. found that in prostate cancer, low expression of SLC14A1 and high promoter methylation may be new indicators for PCa (prostate cancer) progression and prognosis [65]. Additionally, Wang’s analysis in acute myeloid leukemia (AML) revealed that SLC14A1 can act as an endocytosis-related gene (ERG) and N6-methyladenosine (m6A)-related gene, affecting AML progression and metastasis [66].

### 3.3. The Role of UTs in Tumor Metabolic Reprogramming

#### 3.3.1. Urea Metabolism and Mitochondrial Oxidative Stress

Urea metabolism has potential intersections with tumor metabolic reprogramming. A high-urea environment can significantly downregulate early growth response factor 1 (Egr1) while upregulating ERK pathway activity. This regulatory pattern is highly similar to the metabolic adaptive changes commonly observed in tumor cells [67]. UTs play a significant role in this process.

Li et al. reported that overexpression of UT-B in B16 mouse melanoma cells influences polyamine metabolism and the urea cycle, leading to a decrease in cell proliferation and an increase in apoptosis. This alteration significantly modifies the metabolic pathways associated with the urea cycle, decreases urea and polyamine production, and increases nitric oxide production. This could be because the overexpression of UT-B leads to mitochondrial dysfunction and oxidative stress, which in turn activates P53. Consequently, the activated P53 suppresses polyamine metabolism [68]. Other studies reported that SLC14A1 gene downregulation induces the accumulation of mitochondrial reactive oxygen species in A498 cells, decreases the levels of intracellular adenosine triphosphate, while also compromising the integrity of mitochondrial membrane potential and altering mitochondrial morphology.

#### 3.3.2. Amino Acid Metabolism

In UC (urothelial cancer), UT-B could prevent the accumulation of urea and arginine via the mTOR signaling pathway. Knockout of SLC14A1 leads to urea accumulation in bladder urothelial cells, leads to a disruption in the equilibrium between the arginine-ornithine-polyamine metabolic cascade and the arginine-citrulline-nitric oxide signaling pathway. Consequently, urothelial cells exhibit increased vulnerability to genomic instability, which ultimately facilitates the development of bladder malignancies. These findings are consistent with in vitro studies on T24 cells, which exhibited cell cycle delay and apoptosis after urea loading.

UTs are also affected by hypoxic conditions. Under hypoxic conditions, NFAT5 enhances UT-A1 expression in mouse embryonic fibroblast (MEF) cells through hypoxia-induced arginase-2 activation, which elevates ornithine and urea concentrations. Arginase-2 potentially serves as a protective mechanism counteracting detrimental iNOS activity, while UT-A1 upregulation represents a compensatory response to hypoxia-mediated urea overproduction. Shi et al. found that UT-B expression in A498 cells cultured under hypoxic conditions was significantly lower than under normoxic conditions [69].

#### 3.3.3. Participate in Glycolysis and Energy Allocation Strategies

UTs can regulate glucose metabolism. Hexokinase 2 (HK2), a pivotal glycolytic enzyme, catalyzes the phosphorylation of glucose to generate glucose-6-phosphate, representing the initial and rate-limiting reaction in the majority of glucose metabolic pathways [70,71]. Due to HK2’s regulatory role in glycolysis, its upregulation is associated with proliferation, migration, invasion, and stemness [72,73], leading to poor prognosis in solid tumors with high HK2 levels [74,75]. Studies have found that HK2 expression in urothelial carcinoma and NSCLC cells is suppressed by SLC14A1. Studies have found that HK2 expression in urothelial carcinoma and NSCLC cells is suppressed by SLC14A1. Knockdown of SLC14A1 increases HK2 mRNA and protein levels in Calu-6 and A549 cells, thereby enhancing cellular glucose uptake and ATP levels and modulating ECAR, suggesting that SLC14A1 may regulate glucose metabolism and inhibit glycolysis through HK2. Downregulation of SLC14A1 increases aerobic glycolysis, providing sufficient energy to sustain rapid cell proliferation and promote metastasis in NSCLC cells [76]. Another study found that nuclear SLC14A1 recruits HDAC1, transrepresses HK2 and DEGS1, prevents the accumulation of arginine and urea, maintains metabolic homeostasis, promotes mitochondrial fusion and augments oxidative phosphorylation while suppressing glycolytic activity through modulation of key regulatory proteins. Furthermore, it significantly enhances the therapeutic efficacy of arginine deprivation in UC-derived cells with ASS1 deficiency [77].

Abnormal energy metabolism is intricately linked to tumor progression. The interaction of UTs with energy metabolism shows at multiple levels. Firstly, the urea transport process itself consumes energy and is closely associated with ATP metabolism in tumor cells. Secondly, research has found significant differences in the urea/water transport ratios mediated by UT-A2 and UT-A3 [78], and this selective transport characteristic may influence the energy allocation strategies of tumor cells.

### 3.4. UTs Participates in Shaping of the Tumor Immune Microenvironment

Emerging evidence demonstrates a strong association between inflammatory processes and tumorigenesis. Numerous malignancies arise from regions characterized by persistent infections, chronic inflammatory states, or prolonged tissue irritation. Contemporary research has elucidated that the tumor microenvironment is predominantly regulated by inflammatory mediators, playing a crucial role in oncogenesis by facilitating neoplastic cell proliferation, enhancing cellular survival, and promoting metastatic dissemination [79]. UTs have been demonstrated to interact with several inflammatory factors. Studies have shown that UT-B-deficient mice exhibit features such as urea accumulation and disrupted NO production, indicating that UT-B plays a role in modulating inflammatory responses. Its inhibitor (UTBinh-14) significantly attenuates LPS-induced TNFα and IL-6 production in BV2 cells, accompanied by reduced NO release, further supporting this observation [80]. In renal cancer, UT-B suppresses hypoxia-induced inflammatory cytokine release and arrests the cell cycle at the G1/S checkpoint [69].

Cancer-associated fibroblasts (CAFs) represent a heterogeneous population of stromal cells that constitute a fundamental component of the tumor microenvironment. These specialized fibroblasts promote fibrotic processes within the TME through enhanced deposition and structural reorganization of the extracellular matrix., thereby actively hindering anti-tumor immune responses by excluding immune cells [81,82,83,84]. Through single-cell transcriptomic profiling of bladder carcinoma specimens, Ma et al. identified tumor-associated fibroblasts (CAFs) characterized by overexpression of SLC14A1. BC patients exhibiting elevated level of intratumoral SLC14A1^+^ CAFs exhibited poor outcomes, including lower response rates to neoadjuvant chemotherapy or immunotherapy. Activation of the cGAS-STING signaling cascade in neoplastic cells stimulates interferon synthesis, establishing a connection between this pathway and the differentiation of SLC14A1^+^ CAF. Moreover, suppression of SLC14A1^+^ CAF development through STAT1 or STING inhibition enhances tumor cell vulnerability to chemotherapeutic agents [85].

According to a recent MR (Mendelian randomization) analysis, SLC14A1 was identified as a biomarker closely associated with immune cell infiltration of prostate cancer. The results indicated that SLC14A1 was positive association with activated dendritic cells and quiescent natural killer cells, while exhibiting an inverse correlation with resting dendritic cells and M1-polarized macrophages [86].

UT-A can be regulated by inflammatory factors. For example, in renal tubular epithelial cells (TECs), IL-11 stimulates STAT3-mediated downregulation of UT-A [87]. Additionally, SLC14A2 is associated with the tumor immune microenvironment. Li et al. identified SLC14A2 as a potential immune-related gene signature in LUAD and LUSC subtype [88], suggesting that SLC14A2 may serve as a potential target for tumor immunotherapy.

### 3.5. The Correlation Between Expression of UTs and Clinicopathological Features of Tumors, and the Potential of UTs as Tumor Biomarkers

To date, UTs have been found to exhibit significant differences in expression between various tumors and normal tissues (Table 4 and Table 5). In melanoma, the expression of UT-B protein was found to be reduced compared to that in normal tissues [68]. Shen et al. analyzed the causal relationship between plasma metabolites, proteins, and EC through genome-wide association studies (GWAS) and discovered differential expression of SLC14A1 in non-EC and different EC subtypes, which suggests its potential as a diagnostic marker for distinguishing non-EC from EC [89].

There are a series of studies that have reported that urologic neoplasms are associated with SLC14A1. Several studies have reported a remarkable decrease in SLC14A1 expression during PCa progression [65,86]. Ye et al. observed that elevated levels of SLC14A1 expression were associated with prolonged biochemical recurrence-free survival in prostate cancer patients. and they identified SLC14A1 as a novel and important BCR-related gene in PCa [90]. Xiao et al. used WGCNA (weighted gene co-expression network analysis) from GEO databases to identify SLC14A1 as a hub gene in PCa. Additionally, SLC14A1 expression showed significant associations with immune cell dysregulation and drug sensitivity in PCa [91]. In cell line LNCaP, it was found that castration affects SLC14A1 expression [90]. SLC14A1 was reported to be expressed at a low level in almost every type of renal cancer tissues. Single-cell data from KIRC (kidney renal clear cell carcinoma) indicated the predominant expression of SLC14A1 in endothelial cells. Upregulation of SLC14A1 suppressed the proliferation, migration, and metastatic dissemination of renal carcinoma cells, highlighting its critical role in renal cancer progression and potential as a novel biomarker. Kaplan–Meier analysis suggested thatelevated expression level of SLC14A1 were significantly associated with improved overall survival outcomes in RCC patients [69,92,93]. There are quite a number of reports about the relationship between bladder cancer and SLC14A1. Chan et al. identified SLC14A1 protein levels as an independent prognostic factor in UC [77]. Altered SLC14A1 expression in human UC may underscore its importance as a novel research target [25,94].

SLC14A1 was also investigated in lung cancers. Zhou et al. observed downregulation of SLC14A1 in tumor tissues across multiple NSCLC datasets. Knockdown of SLC14A1 promoted proliferation and migration in Calu-6 cells, while overexpression in A549 cells and had the opposite effect, suggesting SLC14A1 as a potential diagnostic marker or therapeutic target for NSCLC [95]. Another investigation in CRC reported that SLC14A1 as a DEG may act as a key regulator in CRC metachronous liver metastasis, with its expression level shows a significant association with both recurrence-free survival and overall survival in individuals diagnosed with colorectal cancer [49].

SLC14A1 has also been used as a stem cell marker. Peñarando et al. found that c-PTIO (NO scavenger) can reduce SLC14A1 mRNA levels in colon cancer tumor-associated stem cells (CSCs) [96].

There is relatively limited research on SLC14A2 in tumors, but it has been identified as a drug sensitivity gene that can be utilized in some anti-tumor drug screening studies. Gao et al., based on analysis of the Genomics of Drug Sensitivity in Cancer (GDSC) database, found that SLC14A2 was one of the nine ultimately identified genes [97]. Zhang et al.’s research revealed that the expression level of SLC14A2 could indicate differences in cisplatin sensitivity between HPV^+^ and HPV^−^ tumors [98]. Additionally, SLC14A2 has been identified as an immune-related gene significantly associated with the prognosis of LUAD [88].

### 3.6. Potential of UTs as Targets for Tumor Therapy

Diuretic drugs targeting UTs have been used clinically for a long time [99]. Recently, UT-B was also reported to be used as a novel therapeutic target for neuroinflammatory diseases [80]. The use of UTs as tumor therapeutic targets is still in the exploratory stage, with few related studies. Firstly, the role of SLC14A1 in CRC metastasis opens a pathway for potential therapeutic innovation. Targeting its interaction dynamics with TβRII could inhibit TGF-β signaling, slow EMT progression, and potentially attenuate the metastatic process. Secondly, c-PTIO can reduce SLC14A1 mRNA levels in CSCs, which is highly important for the exploration of new therapeutic targets for colon cancer. Thirdly, miR-10a-3p might participate in the malignant progression of FLT3-mutated AML by targeting genes such as SLC14A1. This finding suggests that microRNA targeting SLC14A1 may inhibit a specific type of tumor progression. Fourthly, MDM2-mediated ubiquitination and degradation of UT-A1 suppress UT-A1 through the binding of UT-A1 to MDM2. Therefore, developing small molecules to block MDM2-SLC14A2 binding could restore the activity of UT-A1. Fifthly, the involvement of SLC14A1 in hypoxia-driven renal cell carcinoma progression via mitochondrial-dependent pathways implies that oxygen-targeted therapeutic approaches could potentially improve treatment outcomes for this malignancy. As research advances, more mechanisms by which UTs are involved in tumor progression may be discovered, and cancer treatment strategies targeting UTs will gradually be developed to assist in tumor diagnosis and treatment (Table 6).

### 3.7. Research Methods for Urea Transporters in Cancers

Immunohistochemistry (IHC), Western blot and Immunofluorescence technologies allow for the direct observation of the distribution of urea transporters in cancer tissues, enabling the correlation of urea transporter expression with clinical features such as pathological grading and staging of cancer, thereby providing valuable information for clinical diagnosis and prognosis assessment. Simultaneously, these technologies also offer the possibility of subcellular localization of urea transporters, allowing for a clear visualization of their positioning in cancer cells, adjacent tissues, and the tumor microenvironment. Urea transporters may primarily localize to the cell membrane of cancer cells, which is related to their function, but they may also appear in the cytoplasm or nucleus, potentially indicating additional biological functions beyond transport.

Proteomics can focus on the urea transporter protein itself, quantitatively detecting the expression abundance of urea transporter proteins in cancer tissues and their post-translational modifications. Various post-translational modifications, including methylation and glycosylation, can have a substantial impact on the activity, localization, and functionality of urea transporters Identifying other proteins that interact with urea transporters provides important clues for revealing cancer-specific urea transport mechanisms.

Single-cell RNA sequencing technology can determine the expression patterns of urea transporters in different subpopulations of cancer cells and various cell types in the tumor microenvironment, identify cancer cell subpopulations with unique expression characteristics, and reveal differences in the expression of urea metabolism-related genes. This helps to deepen the understanding of metabolic differences and interactions between cells, as well as the role of urea transporters in maintaining the metabolic homeostasis of cancer cells.

By comprehensively utilizing the aforementioned methods, we can conduct thorough and in-depth research on urea transporters in cancer from multiple levels. This approach helps us more accurately elucidate the expression characteristics, functions, and regulatory strategies of urea transport proteins, providing new insights and methods for the diagnosis, treatment, and prognostic assessment of cancer.

### 3.8. Current Research Gaps and the Overall Future Direction

Future research on urea transporters in tumors could focus on in-depth exploration of tissue specificity, redundancy of urea transporters, and potential side effects of targeting urea transporters.

#### 3.8.1. Tissue-Specific Research

In terms of tissue specificity, future research needs to carry out in-depth studies on the expression patterns and functional characteristics of urea transporters in different tumor tissues. At present, we only know that urea transporters are downregulated in most tumor tissues, but the exact regulatory mechanism behind this phenomenon remains unclear. Future research can utilize single-cell sequencing technology to precisely locate and functionally analyze urea transporters in different cell subsets of tumor tissues, and investigate the role of urea transporters in various immune cells, fibroblasts and many other cell types. These cells engage with tumor cells through intricate mechanisms, where urea transporters are likely to be crucial in modulating the exchange of materials and facilitating signal transduction. These studies are expected to provide a theoretical basis for formulating targeted treatment strategies for specific tumor tissues and offer new targets for blocking tumor metastasis.

#### 3.8.2. Redundancy Studies of Urea Transporters

In view of the redundancy of urea transporters, the compensatory mechanisms among different urea transporter isomers should be comprehensively analyzed. Different isomers may exhibit functional overlap, but the specific compensation pathways and molecular mechanisms remain unclear. Future research may involve the construction of gene—edited animal models in which specific urea transporter isomers are knocked out or overexpressed. Then, to observe changes in tumor cell growth, proliferation and metabolism, as well as the impact of these changes on tumor growth and treatment response. In addition, proteomics and metabolomics techniques can be utilized to analyze the interaction networks and metabolic pathways changes among different subtypes, thereby revealing the full picture of the compensation mechanism. Moreover, the influence of urea transporter isomer redundancy on the anti—tumor therapeutic effect under different treatment conditions should also be clarified. A deep understanding of this redundancy and compensation mechanism will help develop combined targeted therapy strategies and overcome the limitations of single—target therapy.

#### 3.8.3. Research on the Side Effects of Targeting Urea Transporters

When considering the potential side effects of targeted urea transporters, we need to conduct a comprehensive assessment of the systemic effects. Meanwhile, there are challenges in developing safe and effective treatment methods. At present, there is limited understanding of the mechanism of action of drugs that enhance urea transporters in the body. Therefore, future research can explore direct or indirect methods to enhance the expression of urea transporters. Techniques such as radioactive tracer technology and magnetic resonance imaging can be used to monitor the distribution and metabolic process of drugs in real time and assess their potential impact on normal tissues and organs. We can establish a preclinical toxicity assessment model to simulate the human physiological environment and observe the toxic reactions of drugs on different organ systems and their regulatory effects on the immune system. This will help to study how to reduce side effects and improve the safety and effectiveness of treatment. The new drug delivery system can be designed to achieve targeted drug delivery and minimize drug exposure in non-target tissues. In addition, through the combination of bioinformatics and artificial intelligence technologies, highly selective and low-toxicity drug molecules can be screened and optimized, providing support for the development of safe and effective targeted therapies.

Future research on urea transporters in tumors will be a multidimensional and systematic process, requiring the comprehensive application of various technical approaches and research methods to deeply explore issues such as tissue specificity, compensation and redundancy among isoforms, and the side effects of targeted therapies. This will provide new insights and approaches for the precise treatment of tumors.

## 4. Conclusions

SLC proteins are closely related to the metabolic processes of organisms and the normal operation of the immune system [100,101,102]. Urea cycle reprogramming is a hallmark of tumor formation [1,3], and UTs play crucial role during this process. It has been found that in various cancers, UTs are abnormally downregulated, leading to the accumulation of urea within cells or an imbalance in osmotic pressure. On one hand, urea in tumor cells participates in the Warburg effect through a unique anabolic pathway, providing additional nitrogen sources and energy for tumor cells. On the other hand, UTs participate in the occurrence, progression, metabolic regulation and inflammation of tumors through the regulation of multiple tumor-related signaling pathways, promoting tumor progression. Given the abnormally low expression of UTs in tumor tissues, it is expected to become an important biomarker for the diagnosis and prognosis of various cancers, especially urinary system tumors. As research advances, more mechanisms by which UTs are involved in tumor progression may be discovered, and cancer treatment strategies targeting UTs will gradually be developed to assist in tumor diagnosis and treatment.

## Figures and Tables

**Table 3:** The mechanistic roles of urea transporters in cancer.

Transporters	Cancers or Cell Lines	Observed Effect	Mechanistic Pathway	References
UT-B (*SLC14A1*)	PCa	High expression of SLC14A1 could increase the BCR-free survival time of PCa patients	① SLC14A1 inhibited the mTOR signaling pathway; ② interaction with miRNAs (has-miR-508, has-mir-514a2, and has-mir-449a) and the infiltration of B cells.	PMID: 36257969
UT-B (*SLC14A1*)	PCa	overexpression of SLC14A1 inhibited cell proliferation and metastasis	① Attributed to hypermethylation on SLC14A1 promoter region. ② SLC14A1 overexpression also suppressed CDK1/CCNB1 pathway and mTOR/MMP-9 signaling pathway.	PMID: 38942821
UT-B (*SLC14A1*)	UBUC/UTUC	SLC14A1 played tumor suppressive roles through the inhibition of cell proliferation and metastasis in distinct UC-derived cells and animal models.	① SLC14A1 prevented the accumulation of arginine and urea; ② Nuclear SLC14A1 transrepressed HK2 and DEGS1 genes via recruitment of HDAC1 and/or SIN3A to maintain metabolic homeostasis and thereafter impeded tumorigenesis; ③ SLC14A1 inhibited the mTOR signaling pathway	PMID: 33052246
UT-B (*SLC14A1*)	KIRC	low levels of SLC14A1 expression were associated with a better clinical prognosis	The abnormal activation of the JAK-STAT signaling pathway	PMID: 37004669
UT-B (*SLC14A1*)	CRC patients with metachronous liver metastasis	markedly upregulated in CRC patients with metachronous liver metastasis	① Upregulated TGF-β/Smad signaling and EMT, amplifying TGF-β/Smad signaling; ② SLC14A1 interacted with TβRII and stabilized TβRII protein; ③ establishing a positive feedback loop	PMID: 39061061
UT-A (*SLC14A2*)	LUAD/LUSC	The expression level is positively correlated with the infiltration of immune cells (CD8^+^, CD4^+^ T cells, macrophages, etc.). High expression indicates a relatively good overall survival	Co-expression with immunomodulatory genes suggests its role in the tumor immune microenvironment	PMID: 34887858

**Table 4:** Studies that report the expression of urea transporters across different cancer types.

Cancers	UTs	Expression in Tumor Tissues Than that in Normal Tissues	Detection Method	Sample Size	References
UBUC/UTUC	*SLC14A1* (UT-B)	Downregulation in UCs	IHC	36 UBUCs + 42 UTUCs with normal	PMID: 33052246
				295 UBUC + 340 UTUC	
			next-generation sequencing	308 and 93 UBUC	
PCa	*SLC14A1* (UT-B)	Significantly reduced in prostate cancer cells and tissue	next-generation sequencing, IHC	429 samples from TCGA + 106 samples from GEO Tissue chip containing 50 prostate cancer patients	PMC9579171
PCa	*SLC14A1* (UT-B)	significantly downregulated in PCa progression	next-generation sequencing,	6 normal prostate tissues, 7 PCa tissues and 6 metastatic PCa tissues from TCGA database.	PMC11213927
KIRC	*SLC14A1* (UT-B)	lowly expressed in renal cancer tissues	RNA-seq, RT-PCR, single-cell transcriptomic data, RT-PCR, Western-blotting and immunohistochemistry.	539 renal cancer specimens and 79 normal renal tissue samples; 63 renal cancer specimens and 14 paracancerous tissue samples; renal clear cell carcinoma cancer tissue (*n* = 9) and paracancerous tissue samples (*n* = 9) from TCGA and GEO	PMID: 37004669
CRC	*SLC14A1* (UT-B)	overexpression significantly correlating with poor relapse-free and overall survival	RNA-seq; IHC	The sample size is unclear; 230 patients, consisting of 128 males and 102 females,	PMC11282742
LUAD/LUSC	*SLC14A2* (UT-A)	downregulated	RNA-seq (TCGA); qRT-PCR	TCGA (LUAD *n* = 515; LUSC *n* = 501)	PMID: 34887858

**Table 5:** Clinical correlations.

UTs	Cancer Type	Clinical Outcome	Biomarker Potential	References
*SLC14A1* (UT-B)	PCa	High expression of SLC14A1 could increase the BCR-free survival time of PCa patients.	SLC14A1 is a novel important gene associated with BCR of PCa	PMID: 36257969 PMID: 38942821
*SLC14A1* (UT-B)	KIRC	upregulation of SLC14A1 expression levels inhibited the proliferation, invasion, and metastatic ability of renal cancer cells.associated with a better clinical prognosis.	has the potential to become a new biomarker for renal cancer	PMID: 37004669
*SLC14A2* (UT-A)	LUAD	Related to LUAD overall survival	As one of Immune-related genes of LUAD, can not only predict survival outcome but also reflect the immune status of lung cancers.	PMID: 34887858
*SLC14A1* (UT-B)	colorectal cancer metachronous liver metastasis	Overexpression significantly correlating with poor relapse-free and overall survival	underscoring their potential as prognostic markers.	PMID: 39061061
*SLC14A1* (UT-B)	Urothelial carcinoma	Total and membranous SLC14A1 played tumor suppressive roles through the inhibition of cell proliferation and metastasis	SLC14A1 protein level was an independent prognostic factor	PMID: 33052246

**Table 6:** Known or potential strategies targeting urea transporters.

Intervention Strategy	Applicable Cancer Types/Cell Lines	Verified Experimental Model	Mechanisms/Pathways of Action	References
inhibitor of DNA methylation (Decitabine)	PCa	PCa cell lines (22RV1, C4-2) were treated with different concentrations of decitabine for 6 days The qRT-PCR and Western blotting assays revealed that the expression of SLC14A1 at both the mRNA and protein level were increased by DCTB treatment	DNA methyltransferase DNMT3B may mediate methylation of the SLC14A1 promoter region and contribute to its low expression	PMID: 38942821
Over express	PCa	In vitro transfection SLC14A1 to C4-2 and 22Rv1 cell lines Colony-forming	promote the expression of UT-B overexpression of SLC14A1 significantly reduced the protein expression of CDK1 and CCNB1	PMID: 38942821
IFN-γ induce	Bladder cancer-associated fibroblasts (CAF)	Human CAF was cultured in vitro and treated with IFN-γ RNA seq shows that SLC14A1 is upregulated Co-culture experiments showed that the self-renewal of tumor stem cells was inhibited	IFN-γ activates SLC14A1 transcription through the JAK/STAT pathway Enhance urea/arginine metabolism and reduce drug resistance of tumor stem cells	PMID: 36459995
HDAC inhibitor (e.g., SAHA) Coordinated upward adjustment	UTUC/UBUC	Cell lines (RT4, T24) + SAHA ChIP qPCR detection revealed that the HDAC1 binding to the SLC14A1 promoter was removed	HDAC1 participates in the silencing of the SLC14A1 promoter, and the inhibitor relieves this inhibition Recovery of SLC14A1 expression, inhibition of HK2 and DEGS1 transcription	PMID: 33052246
Combination of Metabolism and targeting (Arginine deprivation + SLC14A1 activation)	Bladder cancer and kidney cancer with ASS1 deficiency	In vitro Arg deprivation culture + overexpression of SLC14A1 The cell survival rate has dropped by more than 60% The tumor volume in the mouse model was significantly reduced	SLC14A1 promotes the excretion of urea/arginine and reduces the accumulation of Arg within cells Synergistic death with Arg deprivation drugs (ADI PEG20)	PMID: 33052246

## Data Availability

Not applicable.

## Abbreviations

The following abbreviations are used in this manuscript:

SLC14A1	Solute Carrier Family 14 Member 1
UT	Urea Transporter
DEG	differentially expressed gene
